# Morroniside Inhibits H_2_O_2_-Induced Podocyte Apoptosis by Down-Regulating NOX4 Expression Controlled by Autophagy *In Vitro*

**DOI:** 10.3389/fphar.2020.533809

**Published:** 2020-09-23

**Authors:** Xue Gao, Yi Liu, Lin Wang, Na Sai, Yixiu Liu, Jian Ni

**Affiliations:** ^1^ Beijing University of Chinese Medicine, Beijing, China; ^2^ School of Chinese Materia Medica, Beijing University of Chinese Medicine, Beijing, China; ^3^ Tianjin University of Traditional Chinese Medicine, Tianjin, China; ^4^ Dongzhimen Hospital, Beijing University of Chinese Medicine, Beijing, China; ^5^ School of Pharmacy, Inner Mongolia Medical University, Hohhot, China; ^6^ Research Institute of Chinese Medicine, Beijing University of Chinese Medicine, Beijing, China

**Keywords:** podocyte, autophagy, NOX4, morroniside, apoptosis, oxidative stress

## Abstract

Podocyte apoptosis is the common pathological basis for the progression of various kidney diseases. The overexpression of NOX4, a key enzyme involved in oxidative stress, has been proved to participate in the occurrence of podocyte apoptosis. Autophagy is a kind of adaptive response of cells under stress. However, as a “double-edged sword”, the effect of autophagy on apoptosis in different cells and conditions is complex and variable, which has not been fully explained yet. Morroniside, extracted from the traditional medicinal plant *Cornus officinalis*, has remarkable antioxidant and anti-apoptosis effects, and has been proven to inhibit the overexpression of NOX4 in kidney tissue. Therefore, H_2_O_2_ was used in this study to explore the effects of autophagy on podocyte NOX4 overexpression and apoptosis induced by oxidative stress, as well as the protection mechanism of morroniside in podocytes. The results showed that the autophagy activator rapamycin, as well as the autophagy inhibitor chloroquine, could induce podocyte apoptosis cultured in normal condition, and chloroquine could also significantly increase the NOX4 expression. The NOX4 expression and apoptosis rate of podocytes increased after H_2_O_2_ treatment, the expression of LC3-II decreased, and the expressions of p62, mTOR, and p-mTOR increased. The intervention of morroniside and rapamycin improved autophagy activity and inhibited NOX4 overexpression and apoptosis induced by H_2_O_2_. And chloroquine reversed the inhibitory effect of morroniside on NOX4 overexpression and podocyte apoptosis. Taken together, our results suggest that the expression level of NOX4 in podocytes is regulated by autophagy activity. Morroniside can reduce oxidative stress induced podocyte apoptosis by restoring the damaged autophagy flux and inhibit the overexpression of NOX4.

## Introduction

Podocytes, also known as glomerular visceral epithelial cells, attach to the outside of the glomerular basement membrane (GBM) and participate in the formation of the glomerular filtration barrier. Podocyte injury is involved in the development of almost all glomerulopathies, including focal segmental glomerulosclerosis (FSGS), membranous nephropathy, and diabetic nephropathy (DN) (Serrano-Perez et al., 2018; Sever and Schiffer, 2018). Studies have shown that the degree of podocyte injury is consistent with the severity of proteinuria, glomerulosclerosis, and renal dysfunction (Brosius and RJ, 2014). The injury or loss of podocytes can directly cause macroproteinuria and even death in experimental animals (Trohatou et al., 2017), and more and more researches are willing to take podocytes as potential therapeutic target for kidney diseases (Lal and Patrakka, 2018).

Autophagy is a universal cell biology process involving lysosomes that degrades aging or damaged organelles and macromolecular proteins. It plays an important role in maintaining cellular homeostasis in all major types of kidney cells and is also an adaptive response of cells under stress (Feng et al., 2015). Current research suggests that autophagy is an effective self-protection mechanism against apoptosis (Zhao et al., 2019). As different types of programmed cell death, autophagy and apoptosis can be induced by similar factors and inhibit each other through the activities of a series of terminal molecules (Zhang et al., 2016). More interestingly, a time- and concentration-dependent relationship between drug-induced autophagy and apoptosis has been reported: autophagy induced by short-time and low-dose triptolide inhibits apoptosis, while autophagy induced by long-time and high-dose triptolide promotes apoptosis (Zhou et al., 2015). The results indicate that the regulation of autophagy on apoptosis may be diverse and affected by the state of the cells. Therefore, an in-depth study of the regulatory pathways of autophagy on apoptosis is beneficial to better utilize the cytoprotective effect of autophagy and avoid injury caused by excessive autophagy.

Oxidative stress is caused by increased production of reactive oxygen species (ROS) and is a vital pathogenesis of various kidney diseases (Miranda-Diaz et al., 2016; Zhang et al., 2018). Nicotinamide adenine dinucleotide phosphate (NADPH) oxidases (NOXs) are the main enzymes that catalyze the production of ROS, and they transfer electrons from NADPH to molecular oxygen to form O_2_^-^(Rajaram et al., 2018). NOX4 is the major NOXs isoform in kidney, it is mainly expressed in the renal tubular epithelial cells under physiological conditions, while its expression level in podocytes is low (Nlandu Khodo et al., 2012). Abnormally elevated NOX4 expression can induce podocyte injury and loss *via* multiple pathways including hypoxia-inducible factor 1α (HIF1α) and TGF-β (You et al., 2016), and promote podocyte apoptosis (Das et al., 2014). Although it is still unclear whether autophagy has a direct regulatory effect on the expression of NOX4, some studies have shown that the activation of mTOR, the main inhibitory factor of autophagy, can lead to NOX4-induced podocyte loss (Eid et al., 2013; Yang et al., 2018).

Traditional Chinese medicine (TCM) has a natural advantage in maintaining cell homeostasis. A variety of TCM compounds or extracted components have been shown to have autophagy regulation function (Gong et al., 2018; Xu et al., 2018a; Li et al., 2019). Shan zhu yu (*Cornus officinalis*) is one of the most important ingredients in the classic TCM patent prescription “Liuwei Dihuang Wan”. The therapeutic benefit of *C. officinalis* is to “nourish liver and kidney, and achieve astringent essence”. It is one of the most frequently used core ingredients in the TCM treatment of kidney disease. Morroniside is extracted from *C. officinalis* and has remarkable antioxidant and anti-apoptosis functions. Previous research has found that morroniside can reduce kidney damage caused by diabetes and inhibit abnormal increase of NOX4 expression in kidney tissues (Park et al., 2011).

H_2_O_2_ is the primary NOX4-derived ROS in the kidney tissue (Ilatovskaya et al., 2018), so we used H_2_O_2_-treated podocytes in this research to investigate the regulating role of autophagy in oxidative stress-induced NOX4 overexpression and podocyte apoptosis, and the podocyte protection mechanism of morroniside.

## Materials and Methods

### Drugs and Reagents

Morroniside was purchased from Shanghai Yuanye Biotechnology (Shanghai, China). RPMI 1640 medium, fetal bovine serum (FBS), and penicillin-streptomycin were purchased from Thermo Fisher (USA). NOX4 rabbit polyclonal antibody (14347-1), Beclin-1 rabbit polyclonal antibody (11306-1), LC3 rabbit polyclonal antibody (14600-1), Caspase-3 rabbit polyclonal antibody (19677-1), p62 rabbit polyclonal antibody (55274-1), BAX rabbit polyclonal antibody (50599-2), and Bcl-2 rabbit polyclonal antibody (26593-1) were purchased from Proteintech (USA). p22*^phox^* antibody (ab191512) and donkey anti-rabbit IgG H&L (Alexa Fluor 594) pre-adsorbed secondary antibody (ab150068) were purchased from Abcam (UK). mTOR antibody (2972), p-mTOR Ser2448 antibody (2971) were purchased from Cell Signal Technology (CST, USA). LC3β immunofluorescence antibody (sc-271625) were purchased from Santa Cruz (USA). ROS assay kit (KGT010-1) was purchased from Nanjing Kaiji Bio-Technology (Nanjing, China). Annexin V/FITC apoptosis assay kit was purchased from BD (USA).

### Cell Culture and Treatment

Conditional immortal mouse podocyte cell strain MPC-5 was kindly provided by Professor Weijing Liu from Dongzhimen Hospital, Beijing University of Chinese Medicine. Podocytes were cultured in RPMI-1640 medium supplemented with 10% FBS, 100 U/ml penicillin and streptomycin in an incubator including 5% CO_2_ and 95% air. Cells were cultivated at 33°C with 10 U/ml γ-interferon for proliferation, and 37°C without γ-interferon for 10–14 days to be fully differentiated. Podocytes prepared for experiments were serum deprived for 24 h prior to intervention. In order to study the protective effect of morroniside on H_2_O_2_-treated podocytes, the cells were pre-treated with 5 μM or 2.5 μM morroniside for 2 h, and then treated with 15 μM H_2_O_2_ for 24 h. In order to study the effect of podocyte autophagy on NOX4 expression, the cells were pre-treated with 5 μM rapamycin (RAP) or 5 μM chloroquine (CQ) for 2 h in the relevant experiments.

### MTT Assay

Matured podocytes were added into 96-well plates at a density of 4 × 10^3^ cells per well. Cells were cultured for 24 h after intervention, then 90 μl serum-free medium and 10 μl of MTT were added into each well. After another 4 h culture, the optical density (OD) was measured at 490 nm with an enzyme marker and the cell proliferation rate was calculated.

### Apoptosis Analysis

According to the Annexin V/FITC apoptosis assay kit (BD, USA), podocytes were double stained with FITC-Annexin V and propidium iodide (PI). The apoptotic rates were analyzed using a flow cytometer (AN12060) and the CXP 2.3 software.

### Western Blot Analysis

Podocytes were collected and lysed in lysis buffer with protease inhibitor cocktail and phosphatase inhibitor for protein extraction. Equal amount of protein lysates (30 μg) were separated by 12.5% SDS-PAGE and electrotransferred onto nitrocellulose membranes. After being blocked in TBST containing 5% non-fat dry milk for 1 h, the membranes were probed with the primary antibodies separately at 4°C overnight, and then incubated with a horseradish peroxidase-conjugated secondary antibody for 2 h at room temperature. Peroxidase-labeled protein bands were detected by ECL reagents and the protein intensity was quantified with the Image-Pro Plus 6.0 software (Media Cybernetics, Rockville, USA).

### Immunofluorescence

Podocytes were washed with cold PBS three times, fixed in 4% paraformaldehyde at room temperature for 20 min, permeabilized using 0.3% Triton X-100/PBS for 20 min, then blocked with 3% donkey serum at room temperature for 30 min, and incubated with primary antibodies at 4°C overnight. The cells were subsequently incubated with fluorescence-conjugated secondary antibodies for 2 h in the dark at room temperature. Finally, the cells were sealed with neutral resin containing DAPI, and observed with a laser confocal microscope (Leica Microsystems, Germany).

### Transmission Electron Microscopy

Cells were fixed in 2.5% glutaraldehyde for 2 h. After washingwith PBS for 4 times (15 min/each), cells were post-fixed with 1% osmium tetroxide (OsO4) for 2 h, then they were washed with PBS twice (5 min/each) and stained with 2% uranyl acetate for 2 h, dehydrated with acetone, embedded in epoxy resin. Ultrathin sections were observed by using a Transmission Electron Microscopy.

### Statistical Analysis

Data are presented as mean ± standard error (SE). Statistical analysis was performed with GraphPad Prism 7 software (GraphPad Software Inc., San Diego, CA, USA). Comparison among experimental groups was performed using Student’s *t*-test or analysis of variance (ANOVA), with a value of *P <*0.05 being considered as statistically significant.

## Results

### Effect of Morroniside on Proliferation and Apoptosis of Podocytes Subjected to Oxidative Stress Induced by H_2_O_2_

**Figure 1:**
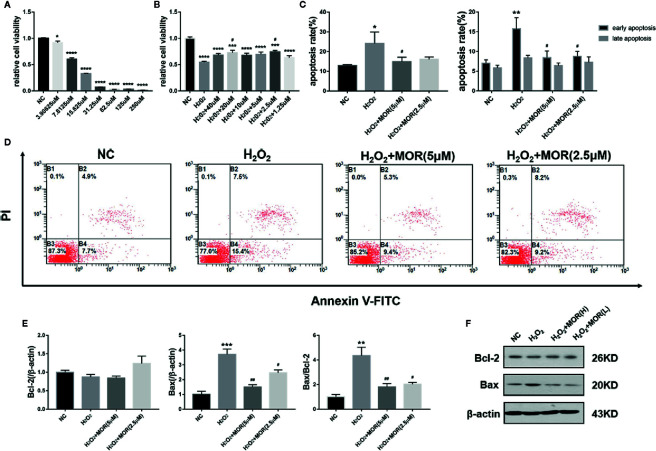
Effect of morroniside on proliferation and apoptosis of podocytes subjected to oxidative stress induced by H_2_O_2_. **(A)** MPC-5 podocytes were treated with different concentrations of H_2_O_2_ for 24 h, and the cell proliferation rate was measured by MTT. **(B)** The H_2_O_2_ treated MPC-5 podocytes were then cultured in complete medium added with different concentrations of morroniside, and the cell proliferation rate was measured by MTT. **(C, D)** Effects of high dose (5 μM) and low dose (2.5 μM) morroniside on the apoptosis rate of MPC-5 podocytes treated with H_2_O_2_. **(E, F)** Effects of high-dose and low-dose morroniside on the expression of bcl-2 and bax in podocytes treated with H_2_O_2_ measured by western blot. n = 3, *p < 0.05, **p < 0.01, ***p < 0.001, ****p < 0.0001 compared to NC; ^#^p < 0.05, ^##^p < 0.01 compared to H_2_O_2_.

We treated MPC-5 podocytes with different concentrations of H_2_O_2_ for 24 h and determined the cell viability by MTT assay. The results showed that from the lowest to the highest concentration, H_2_O_2_ treatment significantly reduced the podocyte viability to various degrees. Considering that the podocyte viability was too low when the H_2_O_2_ concentration was higher than 30 μM, 15 μM was selected as the dose to create oxidative stress (**Figure 1A**).

We then observed the effects of different concentrations of morroniside on the proliferation and apoptosis of H_2_O_2_-treated podocytes. The results showed that morroniside increased the viability (**Figure 1B**) and reduced the apoptosis (**Figures 1C, D**) of H_2_O_2_-treated podocytes. Morroniside also reduced the expression of apoptosis-related proteins bax and reduced the ratio of bax/bcl-2 (**Figures 1E, F**).

### Effect of Morroniside on Autophagy Flux of Podocytes Subjected to Oxidative Stress Induced by H_2_O_2_

**Figure 2:**
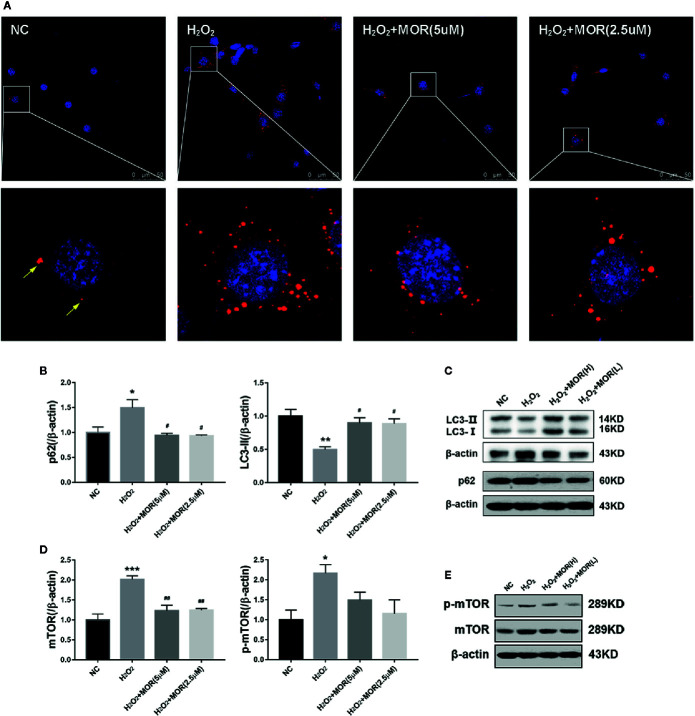
Effect of morroniside on autophagy flux of podocytes subjected to oxidative stress induced by H_2_O_2_. **(A)** P62 expression in podocytes under different conditions was observed by immunofluorescence. **(B, C)** LC3-II and p62 expressions of podocytes under different conditions were detected by western blot. **(D, E)** mTOR and p-mTOR expressions were detected by western blot. n = 3, *p < 0.05, **p < 0.01, ***p < 0.001 compared to NC; ^#^p < 0.05, ^##^p < 0.01 compared to H_2_O_2_.

p62 is an autophagy substrate that is degraded in autophagolysosomes, and accumulates when autophagy flux is impaired (Wang Y. et al., 2018). Therefore, we measured the content of p62 by immunofluorescence and western blot. The results showed that H_2_O_2_ treatment increased the number of p62-positive dots (**Figure 2A**) and p62 protein expression (**Figures 2B, C**) in podocytes.

In the process of autophagy, ubiquitin-like protein LC3-I can be conjugated to PE (and possibly to phosphatidylserine) to form LC3-II, which has a relatively small molecular mass and locates in autophagosome. Therefore, the expression level of LC3-II can be used to evaluate the number of autophagosome and the level of autophagy induction (Klionsky et al., 2012). The results showed that, H_2_O_2_ treatment significantly decreased LC3-II expression in podocytes, and the intervention of morroniside significantly reduced the expression of p62 and increased the expression of LC3-II (**Figures 2A–C**). These results suggest that oxidative stress can induce the inhibition of autophagy in podocytes, and morroniside can restore the damaged autophagy flux.

Mammalian target of rapamycin (mTOR) is the main inhibitory factor of autophagy (Eid et al., 2013). The results showed that H_2_O_2_ treatment significantly increased the expression of mTOR and p-mTOR in podocytes. The intervention of morroniside significantly reversed the change of mTOR, but had no significant effect on the expression of p-mTOR (**Figures 2D, E**).

### Effect of Altered Autophagy Activity on Podocyte Apoptosis and NOX4 Expression

**Figure 3:**
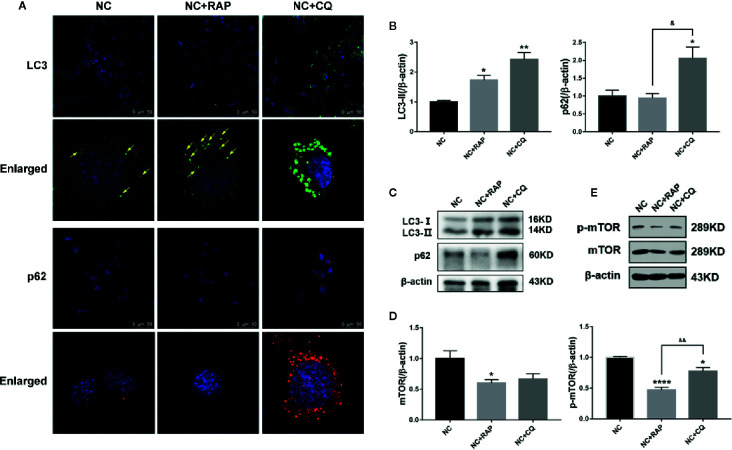
Effects of rapamycin and chloroquine on autophagy flux in podocytes. **(A)** LC3 and p62 expressions of podocytes cultured in normal complete medium with rapamycin (NC + RAP) or chloroquine (NC + CQ) intervention were detected by immunofluorescence. **(B, C)** LC3 and p62 expressions were detected by western blot, n=3. **(D, E)** mTOR and p-mTOR expressions were detected by western blot, n = 3. *p < 0.05, **p < 0.01, ****p < 0.0001 compared to NC; ^&^p < 0.05, ^&&^p < 0.01 compared to NC+RAP.

Rapamycin (RAP) is a commonly used autophagy activator and can inhibit the activation of mTOR; chloroquine (CQ) is an autophagy inhibitor, which can inhibit the fusion of autophagosomes and lysosomes (Klionsky et al., 2012). To investigate the role of podocyte autophagy in regulating the expression of NOX4, we pre-treated the podocytes cultured in normal complete medium for 2 h with rapamycin (NC + RAP) or chloroquine (NC + CQ), then cultured for another 24 h. The results showed that RAP significantly inhibited the expression of mTOR, p-mTOR (**Figures 3D, E**), slightly up-regulated the expression of LC3, but had no significant effect on p62 (**Figures 3A–C**), while CQ significantly up-regulated the expression of LC3 and p62. Interestingly, chloroquine also inhibited the expression of p-mTOR, although the effect was weaker than that of rapamycin, and did not affect the expression level of total mTOR. Some researchers indicated that mTOR signaling could be inhibited during autophagy initiation and be reactivated by the degradation of autolysosomal products (Yu et al., 2010). Therefore, the abnormal accumulation of autophagosomes may be the reason for the decrease of p-mTOR expression induced by CQ.

**Figure 4:**
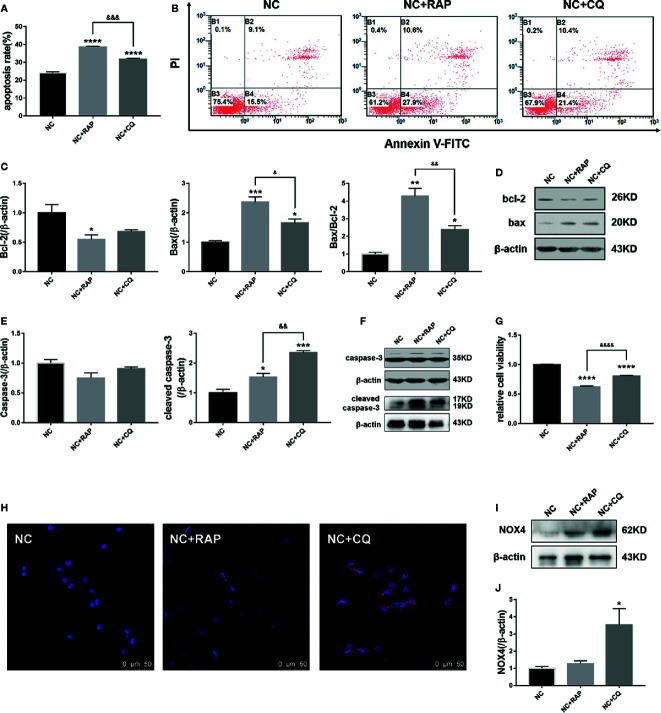
Effect of altered autophagy activity on cell apoptosis and NOX4 of podocytes. **(A, B)** Apoptosis rate of podocytes cultured in normal complete medium with rapamycin (NC + RAP) or chloroquine (NC + CQ) intervention were detected by flow cytometry, n=3. **(C, D)** The expressions of bcl-2 and bax in podocytes cultured in normal complete medium with rapamycin (NC + RAP) or chloroquine (NC + CQ) intervention were detected by western blot, and bax/bcl-2 rate was calculated, n = 3. **(E, F)** The expressions of caspase-3 and cleaved caspase-3 were detected by western blot, n=3. **(G)** The relative cell viability were detected by MTT. **(H)** The expression of NOX4 was detected by immunofluorescence and western blot **(I, J)**, n = 3. *p < 0.05, **p < 0.01, ***p < 0.001, ****p < 0.0001 compared to NC; ^&^p < 0.05, ^&&^p < 0.01, ^&&&^p < 0.001, ^&&&&^p < 0.0001 compared to NC+RAP.

Subsequently, we found that both rapamycin and chloroquine could reduce the proliferation rate of podocytes (**Figure 4G**) and increase the apoptosis rate (**Figures 4A, B**). We detected the expression of apoptosis-related proteins by western blot. As the results shown in **Figures 4C–F**, RAP and CQ had no significant effect on the expression of caspase-3 and bcl-2, and both of them up-regulated the expression of cleaved caspase-3, bax in podocytes to varying degrees, and reduced the bax/bcl-2 ratio, which indicated that autophagy has a “double-edged sword” effect in podocytes, high or low level of autophagy activity could induce podocyte apoptosis.

We also detected the expression of NOX4 by western blot. As the results shown in **Figures 4H–J**, after activating podocyte autophagy, the expression of NOX4 did not show obvious change (NC + RAP vs NC, p > 0.05); However, when the autophagy flux was suppressed, the expression of NOX4 increased significantly (NC + CQ vs NC, p < 0.00001). The above results indicated that autophagy suppression could cause NOX4 overexpression in podocytes, and further promote apoptosis.

### Effect of Altered Autophagy Activity on the Regulation of NOX4 Expression and Apoptosis in H_2_O_2_-Treated Podocytes by Morroniside

The previous results indicated that morroniside can reverse autophagy inhibition and reduce podocyte apoptosis induced by H_2_O_2_. In order to investigate the relationship between the effects of anti-apoptosis and autophagy activity regulation, RAP or CQ was used separately or in combination with 5 μM morroniside to treat the podocytes cultured in complete medium containing 15 μM H_2_O_2_, and the effects on NOX4 expression level was observed.

**Figure 5:**
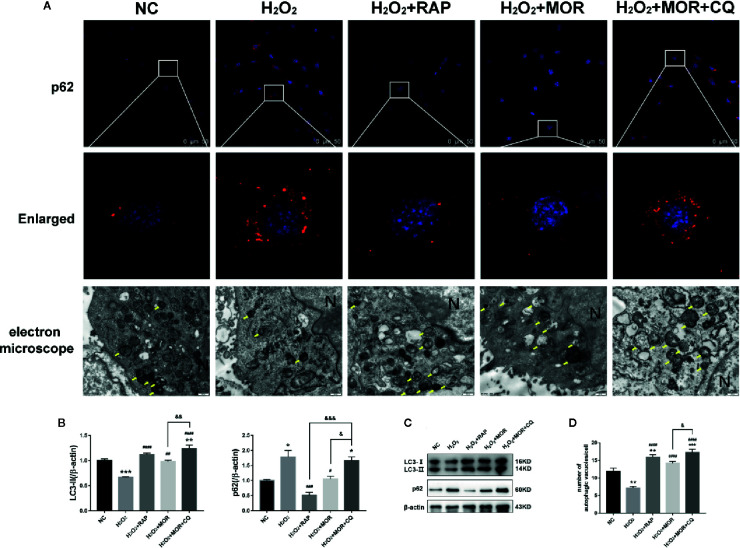
Effects of rapamycin and chloroquine on autophagy flux in morroniside treated podocytes subjected to oxidative stress induced by H_2_O_2_. **(A)** P62 expression in podocytes under different conditions was observed by immunofluorescence. Autophagic vesicles (yellow arrows) in podocytes under different conditions was observed by transmission electron microscopy, bar = 500 nm. **(B, C)** LC3 and p62 expressions were detected by western blot, n = 3. **(D)** Quantification of the autophagic vesicles in 10 randomly selected cells. *p < 0.05, **p < 0.01, ***p < 0.001 compared to NC; ^#^p < 0.05, ^##^p < 0.01, ^###^p < 0.001, ^####^p < 0.0001 compared to H_2_O_2_; ^&^p < 0.05, ^&&^p < 0.01, ^&&&^p < 0.001 compared to H_2_O_2_+MOR.

The expression of p62 and LC3-II was detected by immunofluorescence and western blot, and the number of autophagosome was observed by electron microscope to evaluate the autophagy activity of podocytes. The results showed that morroniside and RAP significantly reduced the expression of p62 in podocyte stimulated by H_2_O_2_, increased the expression of LC3- II (**Figures 5A-C**) and the number of autophagosomes in podocytes (**Figures 5A, D**), and the intervention of CQ reversed the effect of morroniside on p62.

**Figure 6:**
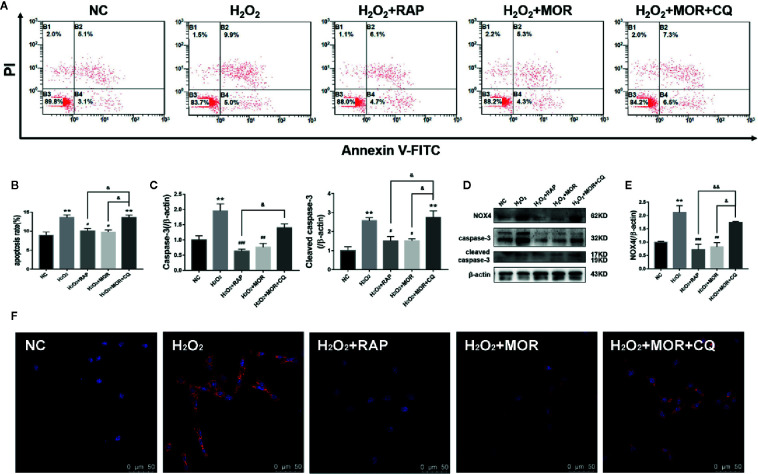
Effect of altered autophagy activity on the regulation of cell apoptosis and NOX4 expression in H_2_O_2_-treated podocytes by morroniside. **(A, B)** Flow cytometry was used to detect the apoptosis rate of podocytes cultured in different conditions. **(C–E)** Caspase-3, cleaved caspase-3 and NOX4 were detected by western blot, n = 3. **(F)** The expression of NOX4 was detected by immunofluorescence. **p < 0.01 compared to NC; ^#^p < 0.05, ^##^p < 0.01, ^###^p < 0.001 compared to H_2_O_2_; ^&^p < 0.05, ^&&^p < 0.01 compared to H_2_O_2_+MOR.

Then we observed the effects of different interventions on podocyte apoptosis and NOX4 expression induced by H_2_O_2_. The results showed that both RAP and morroniside could reduce the apoptosis rate and down-regulate the expression levels of apoptosis related proteins caspase-3 and cleaved caspase-3 (**Figures 6A–E**), and inhibit the overexpression of NOX4 (**Figures 6D–F**). However, the intervention of CQ reversed the effects of morroniside on podocyte apoptosis and NOX4 overexpression. The results confirmed that the inhibitory effect of morroniside on NOX4 overexpression and podocyte apoptosis is related to autophagy activity regulation.

## Discussion

The injury of podocytes is an important feature of progressive glomerular disease (Artelt et al., 2018), and oxidative stress is one of the common pathogenesis of podocyte injury and podocyte loss in kidney disease (Ye et al., 2019). Factors for podocyte injury, such as high glucose, can induce the accumulation of ROS and the occurrence of oxidative stress by promoting overexpression of NOX4 (Fu et al., 2019; Lowe et al., 2019). At present, NOX4 overexpression is considered as one of the symbols of podocyte oxidative stress (You et al., 2016; Xu et al., 2018b). Compared with other NOXs subtypes, NOX4 plays a more influential role in the pathogenesis of kidney disease. Studies have found that NOX4 gene knockout can reverse kidney damage caused by pathogenic factors. Jha (Jha et al., 2014) *et al*. found that albuminuria levels were significantly reduced in NOX4^-/-^ApoE^-/-^mice compared with wild-type diabetic mice, but this change was not observed in NOX1^-/-^ApoE^-/-^mice. In another study, researchers found that, upon induction of type 1 diabetes with streptozotocin, Nox4 knockout rats exhibited significantly lower basal intracellular Ca(2+) levels in podocytes and less DKD-associated damage than wild type rats did (Ilatovskaya et al., 2018). The overexpression of NOX4 can activate bax (Li et al., 2019) or caspase-3 (Eid et al., 2010) through tumor suppressor transcription factor p53, and induce podocyte apoptosis. The results in this study showed that H_2_O_2_ treatment can also cause the overexpression of NOX4, suggesting that there may be a mutually promoting cycle between NOX4 and ROS under pathological conditions that constantly aggravates podocyte injury and podocyte loss.

Morroniside is an iridoid glycoside derived from *C. officinalis*, and it has been found to have kidney protection function in previous pharmacological studies (Yamabe et al., 2007a; Yamabe et al., 2007b). It has been reported that morroniside could inhibit the overexpression of NOX4 and p22*^phox^*; reduce the expression of ROS and proteins related to lipid peroxidation and oxidative stress, such as Nrf2, HO-1, NF-κB, COX-2, and iNOS; and reduce the expression of apoptosis-related proteins, such as bax and cytochrome C (Park et al., 2011). *In vitro* experiments have confirmed that morroniside has a certain protective effect on mesangial cells (Lv et al., 2016). However, there was still no evidence to prove whether morroniside could prevent podocyte injury. The results of our study showed the inhibitory effects of morroniside on NOX4 overexpression and podocyte apoptosis caused by H_2_O_2_.

As terminally differentiated cells, podocytes have limited proliferative capacity, therefore, the mechanisms of self-renewal and material circulation represented by autophagy are crucial for homeostasis maintenance of podocytes (Wu et al., 2018). The autophagy activity of podocytes has a bidirectional change under different injury conditions. For example, in patients with diabetes (Wu et al., 2018; Zhao et al., 2018; Li et al., 2020) and IgA nephropathy (Liang et al., 2018), decreased podocyte autophagy activity is observed; while in systemic lupus erythematosus (SLE) (Jin et al., 2018) or adriamycin-induced models of nephrotic syndrome (Yu et al., 2018), podocyte autophagy activity is found to be increased. Yu et al. (2019) observed an increase in the number of autophagosomes and the expression of autophagy-related proteins such as LC3B-II and Beclin-1 in podocytes stimulated by lupus autoantibodies. In the present study, the expression of LC3-II and the number of autophagosomes decreased after H_2_O_2_ treatment, suggesting that the formation of autophagosomes was blocked. We also found that H_2_O_2_ treatment caused p62 accumulation in podocytes, suggesting that autophagy flux was impaired. However, the intervention of morroniside reversed the above changes in autophagy activity. In addition, we used rapamycin (an autophagy activator) and chloroquine (an autophagy inhibitor) to interfere with podocytes cultured in normal conditions. The results showed that both chloroquine and rapamycin could up-regulated the LC3 expressions in podocytes, and chloroquine had a stronger elevating effect on LC3 than rapamycin and could inhibit the degradation of p62 at the same time. Overall, our findings suggested that the evaluation of autophagy activity requires multiple perspectives. mTOR is the main inhibitor of autophagy. mTOR complex 1 (mTORC1) can prevent the formation of ULK1 complex by phosphorylating ULK1, thus inhibiting the formation of autophagosomes (Bork et al., 2019). In the present study, the expression levels of mTOR and p-mTOR were significantly increased by H_2_O_2_, suggesting that H_2_O_2_-induced autophagy inhibition may be related to the excessive activation of mTOR. Further, the intervention of morroniside reduced total mTOR expression but had no obvious effect on mTOR phosphorylation.

Most studies suggest that autophagy plays a protective role in podocytes (Ye et al., 2019), but some scientists hold different views. Su et al. (2020) have demonstrated that autophagy is involved in podocyte apoptosis caused by tumor necrosis factor-α (TNF-α), and some IncRNAs can exert anti-apoptotic effects *via* inhibiting autophagy. It is remarkable that podocyte apoptosis and autophagy activation often appear together. Wang et al. (2018) found that when the *NUP160* gene was knocked out, podocyte apoptosis increased, and autophagy was activated simultaneously. However, these findings did not confirm that autophagy is necessarily a factor of podocyte injury, or that excessive autophagy occurs during podocyte injury. In the present study, both autophagy suppression and overactivation promoted apoptosis of podocytes, although overactivation of autophagy did not cause NOX4 expression fluctuation, suggesting that NOX4 was partially involved in autophagy regulation of apoptosis. The results also confirmed the “bidirectional regulation” of autophagy on apoptosis. Rubinstein (Rubinstein and Kimchi, 2012) et al. proposed that activation of autophagy might reflect a crosstalk between the two processes. Autophagy would not restore to the baseline levels if the stress persists, and no longer support cell survival, cells autophagy might respond by activating apoptosis in order to ensure efficient elimination of damaged cells, without triggering local inflammation.

As mentioned above, autophagy is an adaptive response of cells under stress (Feng et al., 2015), therefore, autophagy may be activated as a protection to injury. Matsuda et al. (2018) found that glomeruli of 4-week-old *atg5* conditional knockout mice exhibited slightly distended capillary loops accompanied by an accumulation of ROS, and that the administration of N-acetyl-l-cysteine, a ROS scavenger, could rescue the glomerular phenotypes. In the present study, rapamycin-activated autophagy (presented as an increase in LC3 expression) did not up-regulated the level of NOX4 expression in podocytes cultured in normal medium; while in contrast, inhibition of autophagy by chloroquine resulted in NOX4 overexpression. In addition, the effect of rapamycin also reversed the overexpression of NOX4 induced by H_2_O_2_.

The results above indicate that autophagy is involved in the regulation of oxidative stress. When co-administered with chloroquine, the inhibitory effect of morroniside on NOX4 overexpression and cell apoptosis induced by H_2_O_2_ was reversed. These results suggested that morroniside inhibited NOX4 overexpression and ROS accumulation by restoring blocked podocyte autophagy flux, and reduced podocyte apoptosis in sequence.

## Conclusion

In summary, our study demonstrated that oxidative stress inhibited podocyte autophagy activity, caused NOX4 overexpression and podocyte apoptosis. Morroniside prevented podocyte apoptosis by restoring the blocked autophagy flux and inhibiting the overexpression of NOX4 induced by H_2_O_2_.

## Data Availability Statement

All datasets generated for this study are included in the article/supplementary material.

## Author Contributions

All authors contributed to the article and approved the submitted version. XG and JN conceived and designed the experiments. XG, YL, LW, NS, and YXL performed the experiments. XG and YL analyzed the data. XG wrote the paper.

## Funding

This research was funded by China Postdoctoral Science Foundation, grant number 2019M650595.

## Conflict of Interest

The authors declare that the research was conducted in the absence of any commercial or financial relationships that could be construed as a potential conflict of interest.
